# FVIIa-sTF and Thrombin Inhibitory Activities of Compounds Isolated from *Microcystis aeruginosa* K-139

**DOI:** 10.3390/md15090275

**Published:** 2017-08-30

**Authors:** Andrea Roxanne J. Anas, Akane Mori, Mineka Tone, Chiaki Naruse, Anna Nakajima, Hirohiko Asukabe, Yoshiaki Takaya, Susumu Y. Imanishi, Tomoyasu Nishizawa, Makoto Shirai, Ken-ichi Harada

**Affiliations:** 1 Faculty of Pharmacy, Meijo University, Tempaku, Nagoya 468-8503, Japan; 120973255@ccalumni.meijo-u.ac.jp (A.M.); 110973437@ccalumni.meijo-u.ac.jp (M.T.); 100973149@ccalumni.meijo-u.ac.jp (C.N.); g0973345@ccalumni.meijo-u.ac.jp (A.N.); asukabe@bj8.so-net.ne.jp (H.A.); ytakaya@meijo-u.ac.jp (Y.T.); susima@meijo-u.ac.jp (S.Y.I.); 2 College of Agriculture, Ibaraki University, Ami, Ibaraki 300-0393, Japan; tomoyasu.nishizawa.agr@vc.ibaraki.ac.jp (T.N.); makoto.shirai.kanoha@vc.ibaraki.ac.jp (M.S.); 3 Graduate School of Environmental and Human Sciences, Meijo University, Tempaku, Nagoya 468-8503, Japan

**Keywords:** fVIIa-sTF inhibitors, *Microcystis*, blood coagulation cascade, LC-MS, aeruginosin K-139, thrombin, micropeptin K-139, microviridin B

## Abstract

The rise of bleeding and bleeding complications caused by oral anticoagulant use are serious problems nowadays. Strategies that block the initiation step in blood coagulation involving activated factor VII-tissue factor (fVIIa-TF) have been considered. This study explores toxic *Microcystis aeruginosa* K-139, from Lake Kasumigaura, Ibaraki, Japan, as a promising cyanobacterium for isolation of fVIIa-sTF inhibitors. *M. aeruginosa* K-139 underwent reversed-phase solid-phase extraction (ODS-SPE) from 20% MeOH to MeOH elution with 40%-MeOH increments, which afforded aeruginosin K-139 in the 60% MeOH fraction; micropeptin K-139 and microviridin B in the MeOH fraction. Aeruginosin K-139 displayed an fVIIa-sTF inhibitory activity of ~166 µM, within a 95% confidence interval. Micropeptin K-139 inhibited fVIIa-sTF with EC_50_ 10.62 µM, which was more efficient than thrombin inhibition of EC_50_ 26.94 µM. The thrombin/fVIIa-sTF ratio of 2.54 in micropeptin K-139 is higher than those in 4-amidinophenylmethane sulfonyl fluoride (APMSF) and leupeptin, when used as positive controls. This study proves that *M. aeruginosa* K-139 is a new source of fVIIa-sTF inhibitors. It also opens a new avenue for micropeptin K-139 and related depsipeptides as fVIIa-sTF inhibitors.

## 1. Introduction

Bleeding and bleeding complications are drawbacks of oral anticoagulant use caused by warfarin and other anticoagulants [1,2]. Strategies that block the initiation step and lead to thrombin arrest are new approaches in anticoagulant research and development [3]. Inhibitors of activated fIX (fIXa) and activated fX (fXa) and thrombin have blocked fibrin formation and fibrin-mediated feedback activation [3]. Due to the disadvantages of existing drugs in the market such as heparin and warfarin, various researchers [3,4,5] have designed different strategies for blocking activated fVII (fVIIa) and thrombin. Oral anticoagulants directly inhibit fXa and fIXa. Ximelagatran has a similar activity to warfarin and heparin. Moreover, it has an effect on the elevation of transaminase level [5]. Another strategy for blocking the initiation of coagulation via extrinsic pathway is inhibiting thrombin formation. This approach focuses on blocking the activated factor VII-Tissue Factor (fVIIa-TF) formation and thrombin inhibition as the initial step of coagulation. The fVIIa is the starting enzyme that triggers coagulation in the extrinsic pathway. When coupled with the tissue factor (TF), fVIIa-TF-triggers an initial coagulation leading to fXa, thrombin and finally fibrin clot [6,7,8,9].

The toxic cyanobacterium *Microcystis* found in algal blooms contain toxic microcystins, cyclic depsipeptides or peptides and non-toxic linear peptides [10,11]. Linear peptides from toxic *Microcystis* show serine protease inhibiting activities, which could be of use as anticoagulants in the blood coagulation cascade [2,12]. The cyclic depsipeptides/peptides [13] and linear peptides [14,15,16] from a cyanobacteria origin have been noted to contain serine protease inhibiting activities. Aeruginosin is a class of peptide from cyanobacteria first isolated by Murakami et al. [17]. It is composed of four subunits made of Hpla (hydroxyphenyllactic acid), Leu (leucine), Choi (2-carboxy-6-hydroxyoctahydroindole), and arginine or its derivatives. Aeruginosins—89, 102, 103 [14,18,19,20]—have established thrombin inhibitory activities. Hanessian’s group [20] has further studied the chemistry and serine protease inhibitory activities of aeruginosin. Micropeptin is a class of Ahp-containing cyclic depsipeptides first isolated by Okino et al. [21] from *Microcystis aeruginosa*. Micropeptins A and B [21] are plasmin and trypsin inhibitors at μM concentrations. Also, micropeptins C-F [22] have chymotrypsin inhibitory activities at μM concentration. Microviridin has been first isolated by Kaya’s group [23] from *M. viridis* NIES-102. It inhibits tyrosinase at mM concentration. *M. aeruginosa* K139 has been initially collected from an algal bloom in Lake Kasumigaura in Ibaraki, Japan [24]. The axenic and toxic culture has caused liver damage in mice, with LD_50_ of 7.3 mg/kg [24]. Different studies by Nishizawa et al. [25,26] have identified non-ribosomal peptide synthetase genes for the micropeptin biosynthesis [27]. Various compounds from *M. aeruginosa* K139 have been isolated [24,25,26]. To date, aeruginosin K139, micropeptin K139, microviridin B, some microcystins have been reported [25,26]. From our previous paper [12], we have determined the fVIIa-sTF inhibitors from fifty *M. aeruginosa* strains using liquid chromatography-mass spectrometry (LC-MS), which includes *M. aeruginosa* K139. In this study, we will explain the fVIIa-sTF and thrombin inhibitory activities of compounds isolated from *M. aeruginosa* K139. We have isolated and assayed the fVIIa-sTF and thrombin inhibitors present in the cyanobacteria above and compare their half maximal effective concentrations (EC_50_) values. Also, we will explain the complete structure elucidation of aeruginosin K139 using 1D- and 2D-NMR techniques.

## 2. Results and Discussion

### 2.1. Isolation of Aeruginosin K139, Micropeptin K139, and Microviridin B from M. aeruginosa K139

The active compounds from *M. aeruginosa* K139 underwent acidification with 5% CH_3_COOH to avoid undesirable pigments during isolation. We used 20% MeOH, 60% MeOH, and MeOH as eluents in ODS-SPE to efficiently separate aeruginosin K139 from micropeptin K139. Both compounds contained an arginine/arginine-derived moiety and co-eluted in 60% MeOH. Indeed, we were able to separate and sort out aeruginosin K139 from micropeptin K139 (Supplementary Materials 1). The aeruginosin K139 was eluted first with 60% MeOH with no trace of the latter (Supplementary Materials 2 and 3). Further purification using conventional column chromatography (3 times) afforded aeruginosin K139.

**Figure 1:**
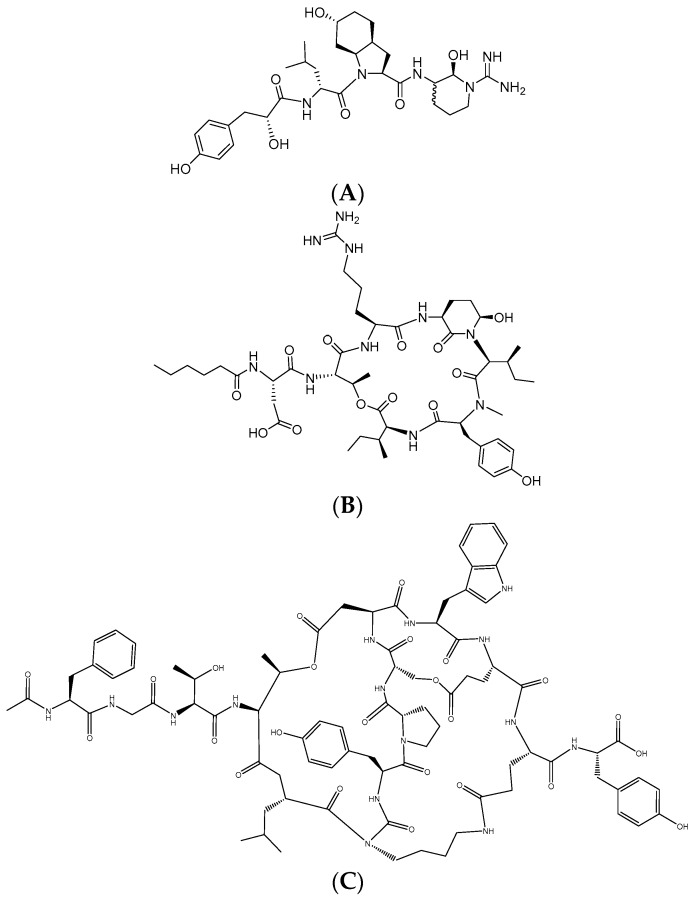
FVIIa-sTF and thrombin inhibitors isolated from toxic *M. aeruginosa* K139. (**A**) aeruginosin K139: EC_50_, µM: thrombin, 0.66; fVIIa-sTF, ~166. Reproduced with permission from Nakano and Harada, Study on non-ribosomal peptide synthesis of peptides by cyanobacteria, BS Thesis; Meijo University, 2003 [30]; (**B**) micropeptin K139:EC_50_, µM: thrombin, 26.94; fVIIa-sTF, 10.62. Reproduced with permission from Nishizawa et al. Characterization of the locus of genes encoding enzymes producing heptadepsipeptide micropeptin in the unicellular cyanobacterium *Microcystis*. The Journal of Biochemistry; Oxford University Press, 2011 [25]; and from Nakano and Harada, Study on non-ribosomal peptide synthesis of peptides by cyanobacteria, BS Thesis; Meijo University, 2003 [30]; (**C**) microviridin B: EC_50_, µM: thrombin, 4.58; fVIIa-sTF, no activity. Reproduced with permission from Nakano and Harada, Study on non-ribosomal peptide synthesis of peptides by cyanobacteria, BS Thesis; Meijo University, 2003 [30].

Micropeptin K139 was co-eluted with microviridin B in MeOH ODS-SPE fraction. The MeOH fraction contained both microviridin B and micropeptin K139 with the ion *m/z* 871 [(M + H_2_O) + 2H]^2+^ and an observed ion at *m/z* 1723 [M + H]^+^ for microviridin B, and *m/z* 987 [M + H]^+^ for the latter. We verified the presence of two compounds in normal thin layer chromatography (TLC) (Supplementary Materials 12). The isolation afforded 3.46 mg of micropeptin K139 after three consecutive open column chromatographies with 65:25:10 CHCl_3_: MeOH: H_2_O as an eluent. Micropeptin K139, with an observed *m/z* 987.57 [M + H]^+^, was commonly found in the *M. aeruginosa* K139 species of cyanobacteria. However, micropeptin K139 observed a similar [M + H]^+^ as micropeptin A [21]. Since both compounds—micropeptin A and K139—have similar [M + H]^+^, in the literature [21,28] extensive discrimination and analysis were done to know the real identity of the isolate. Micropeptin A, with *m/z* 987.57 [M + H]^+^, isolated by Okino et al. [21] has Leu, Val, and Lys moieties while micropeptin K139, isolated by Harada et al. [28], has Ile and Arg. Also, micropeptin A [21] has been reported to be inactive in thrombin inhibitory assay. Our isolate exhibited a thrombin activity with EC_50_ of 26.94 μM. The MS and MS/MS data of micropeptin K139 (Supplementary Materials 13b–d) matched with the MS/MS spectrum of the compound detected by Lombardo et al. [29]. Lombardo’s group [29] deduced peaks at *m/z* 987, 969, 774 and 756. Moreover, the ^1^H-NMR spectrum in DMSO-*d*_6_ (Supplementary Materials 13a) of the isolate proved to be identical with the previously isolated compound of Nakano and Harada [30]. Simultaneously, we have isolated microviridin B together with micropeptin K139 (Supplementary Materials 1, 13a–d and 14a–c and Figure 1). Microviridin B was eluted after micropeptin K139. The isolation afforded 2.55 mg of the above compound. The ^1^H-NMR spectrum in CD_3_OD (Supplementary Materials 14a) of microviridin B matched with the isolate of Nakano and Harada [30]. We also ran the compound in DMSO-*d*_6_ (Supplementary Materials 14b) to find the lost signals or exchangeable hydrogens in CD_3_OD.

### 2.2. Aeruginosin K139

#### 2.2.1. Structure Elucidation of Aeruginosin K139

**Figure 2:**
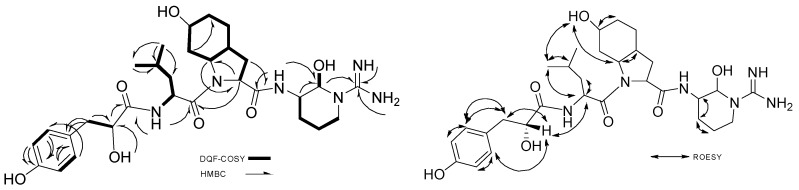
2D-NMR correlations of aeruginosin K139.

Complete carbon and hydrogen assignments of aeruginosin K139 are tabulated in Table 1. Signals for carbon were analyzed and assigned from ^13^C-NMR (Supplementary Materials 6) and HSQC data (Supplementary Materials 7). The signals from 120 ppm to 175 ppm were identified by C12-DMSO-*d*_6_ in HMBC. Exchangeable hydrogens from hydroxyls cannot be seen from the 2D NMR. Complete 2D-NMR correlations of aeruginosin K139 are found in Figure 2.

#### 2.2.2. Stereochemistry of Aeruginosin K139

##### Hpla

The stereochemistry of 4-hydroxyphenyllactic acid (*p*-Hpla) was deduced by comparing the literature values of the ^1^H- and ^13^C-NMR. The stereochemistry of Hpla in aeruginosin K139 was found to be an l-configuration by comparing the literature values of Anas et al. [12] and Vegman and Carmeli [31] for l-Hpla, and Ishida et al. [18] for d-Hpla.

##### Leu

**Figure 3:**
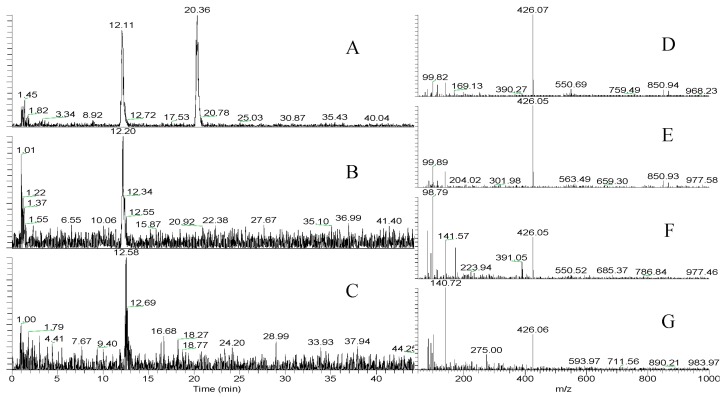
Advanced Marfey analysis of Leu in aeruginosin K139 using LC-MS in 25% acetonitrile with 0.1% formic acid to 65% acetonitrile with 0.1% formic acid over 45 min, TSKgel SuperODS (TOSOH) 100 × 2.0 mm, capillary temp 250 °C, 25 µL injection of 1 mg/mL. (**A**). Extracted Ion Chromatogram (EIC) with *m/z* 426–427 of dl-Leu-l-FDLA; (**B**). EIC of l-Leu-l-FDLA; (**C**). EIC of aeruginosin K139-l-FDLA; (**D**). *m/z* of dl-Leu-l-FDLA with a retention time (t*_R_*, min) 12.1; (**E**). *m/z* of dl-Leu-l-FDLA with a retention time (t*_R_*, min) 20.3; (**F**). *m/z* of l-Leu-l-FDLA with a retention time (t*_R_*, min) 12.2; (**G**). *m/z* of aeruginosin K139-l-FDLA with a retention time (t*_R_*, min) 12.5.

The stereochemistry of Leu was elucidated using advanced Marfey’s analysis [32]. Advanced Marfey’s [32] utilized LC-MS in comparison with Marfey’s, which uses high performance liquid chromatography (HPLC) techniques [33]. The configuration of Leu was found to be l-Leu as compared with authentic samples (Figure 3).

##### Choi

**Figure 4:**
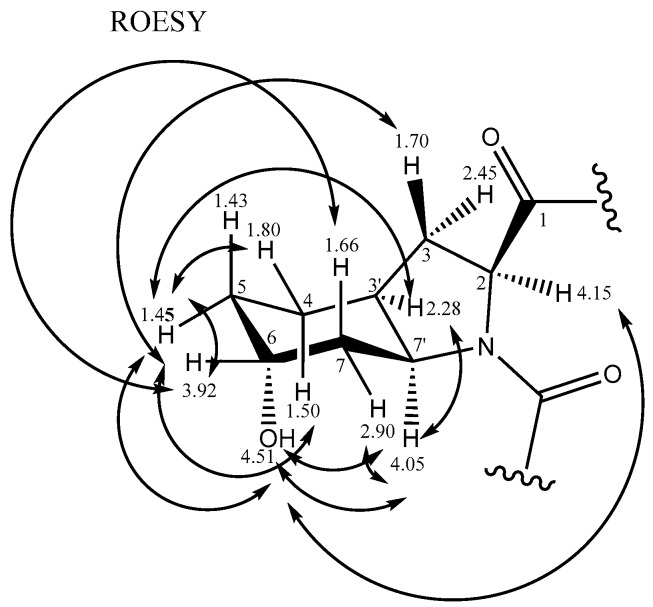
Rotational-frame nuclear Overhauser Effect SpectroscopY (ROESY) correlation of Choi.

The relative stereochemistry of Choi was elucidated using Rotational-frame nuclear Overhauser Effect SpectroscopY (ROESY) data (Figure 4)—H3’ (2.28 ppm) is correlated with H7’ (4.05 ppm); H7’ (4.05 ppm)–H7a (2.90 ppm), H7 (1.66 ppm)–H6 (3.92 ppm). There was no ROESY connectivity between H7’ (4.05 ppm) and H6 (3.92 ppm). This result coincides with the data of l-Choi on aeruginosins LH650A and LH650B, by Vegman and Carmeli [31], with slight chemical shift differences in ^13^C- and ^1^H-NMR. The relative configuration of Choi was established as 2S*, 3aS*, 6R*, 7aS* (l-Choi).

##### Argininal

**Figure 5:**
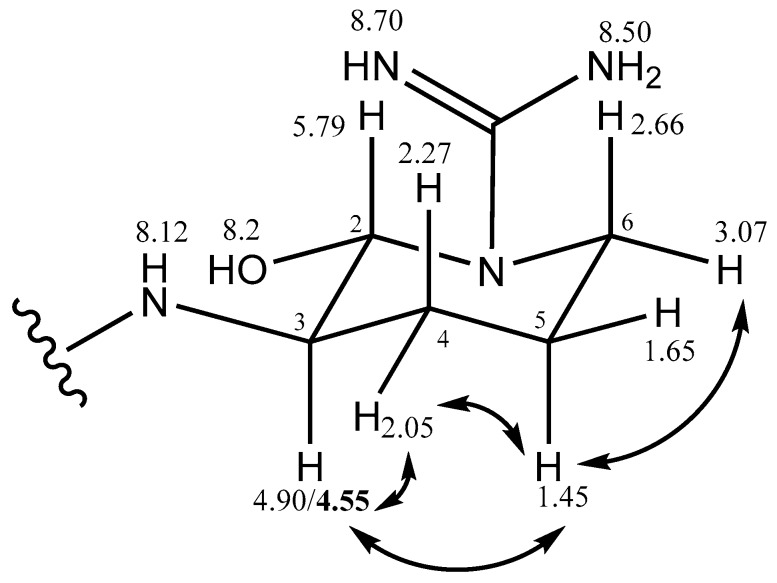
ROESY correlation of argininal in hemiaminal form.

The relative stereochemistry of argininal in hemiaminal cyclic form was elucidated using ROESY data (Table 1 and Figure 5) following the procedure of Kodani et al. [19] for the stereochemistry of 1-amino-2-(*N*-amidino-Δ^3^-pyrrolinyl) ethyl (*Aeap*), and in comparison of chemical shifts from the existing literature. The H3-4.55 ppm is correlated via ROESY to H5a-1.45 ppm, and H5a is associated to H6b-3.07 ppm. We cannot find a ROESY correlation between hydroxyl at C2 and H3. We deduced the structure of the argininal to be l-configuration.

#### 2.2.3. LC-MS and HR-MS of Aeruginosin K139

A possible tautomerization has been observed in aeruginosin K139. This was verified in LC-MS (Supplementary Materials 3) with four dominant peaks having an *m/z* 603. The *m/z* 635 observed in the spectra has been attributed to the MeOH attached to the compound above during the isolation process.

**Figure 6:**
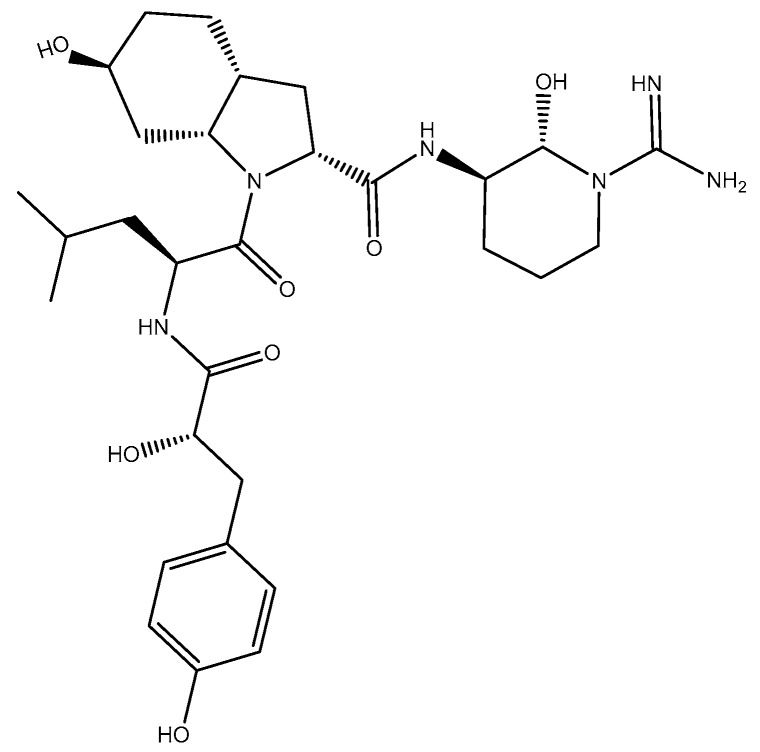
The structure of aeruginosin K139: l-Hpla-l-Leu-l-Choi-l-Argal.

HR-MS of aeruginosin K139 gave an ion with *m/z* 603.3516 [M + H]^+^ (C_30_H_47_N_6_O_7_, Δm = +1.5 mmu, 2.4 ppm). The compound has 11 degrees of unsaturation attributed to 7 double bonds and 4 ring systems composed of phenyl, Choi, and argininal (Figure 6).

### 2.3. FVIIa-sTF and Thrombin Assays

The compounds isolated from *M. aeruginosa* K139 were subjected to fVIIa-sTF and thrombin assays at 10 μg/mL and 100 μg/mL. All three compounds—aeruginosin K139, micropeptin K139, and microviridin B—inhibited thrombin at low and high doses. The micropeptin K139 gave a favorable fVIIa-sTF activity at 10 μg/mL and 100 μg/mL with inhibitory activity greater than 50% and 85%, respectively. Aeruginosin K139 displayed an active fVIIa-sTF inhibitory activity at 100 μg/mL while microviridin B failed to exhibit any activity in fVIIa-sTF assays. The EC_50_ of each compound was computed by GraphPad Prism 7 [34], with 95% confidence. Microviridin B was more explicit in thrombin, with EC_50_ 4.58 μM (Table 2), than other isolates from *M. aeruginosa* K139. However, literature data by Okino et al. [35] presented a negative activity of microviridin B in the thrombin inhibitory assay. A difference in activity for the same compound may be attributed to the different cyanobacterial strains used in the study. Our microviridin B was isolated from the strain of *M. aeruginosa* K139. Okino’s group [35] isolated the compound from the NIES-102 cyanobacterial strain. Micropeptin K139 revealed a favorable fVIIa-sTF inhibitory activity, with an EC_50_ of 10.62 μΜ. Among the three compounds isolated from *M. aeruginosa* K139, micropeptin K139 proved to be the most effective as an fVIIa-sTF inhibitor, with a thrombin/fVIIa-sTF EC_50_ ratio greater than one (Table 2). Although not an fVIIa-sTF specific, it proved to be a more efficient fVIIa-sTF inhibitor than as a thrombin inhibitor. The fVIIa-sTF and thrombin inhibitory assays confirmed that the aeruginosin K139 is more of a thrombin inhibitor than an fVIIa-sTF inhibitor with thrombin EC_50_ 0.66 μM (Table 2). We used ethanol or water as negative controls.

The micropeptin K139 inhibitory activities in fVIIa-sTF and thrombin are comparable with leupeptin and more potent than 4-amidinophenylmethanesulfonylfluoride (APMSF, Wako) (Table 2). We were able to compute a reasonable thrombin/fVIIa-sTF EC_50_ ratio of 2.54. A large thrombin/fVIIa-sTF EC_50_ ratio [36] would indicate a high selectivity against thrombin. The thrombin/fVIIa-sTF EC_50_ ratio of micropeptin K139 was almost twice more than that of leupeptin.

From our search, there have not been any micropeptin K139 serine protease inhibitory studies in the literature. We think it is good to explore this compound, which could lead to a new avenue of anticoagulant study. Related compounds of micropeptin K139 like micropeptins C to F have been isolated by Kisugi and Okino [22] inhibited chymotrypsin with EC_50_ values of 1.1, 1.2, 1.0 and 1.5 μg/mL, respectively. There is no report of thrombin and fVIIa-sTF inhibition from the compounds above. Micropeptin A isolated by Okino et al. [21] does not inhibit thrombin. The presence of arginine in micropeptin K139 makes it a more dominant thrombin inhibitor than micropeptin A.

Micropeptin K139 and aeruginosin K139 were both isolated from the same cyanobacterium *M. aeruginosa* K139. Both compounds contain an arginine or arginine-derived moiety. The possible tautomerization (Supplementary Materials 3) in aeruginosin K139, leading to the formation of a hemiaminal *Aeap* derivative, might be the reason for its weaker fVIIa-sTF activity and a stronger thrombin inhibitory activity. Similar *Aeap* backbone has been observed in aeruginosin 103 [19] from *M. viridis*, and also inhibited thrombin at 9.0 µg/mL. The micropeptin K139 contains linear arginine moiety, which could cling directly to the fVIIa-sTF complex. Thus, it is more active than aeruginosin K139. It is also considered to be a cyclic depsipeptide. At first, we thought that the Ahp-containing moiety in micropeptin K139 was an active moiety in fVIIa-sTF. However, we have tested the three aeruginopeptins—aeruginopeptins 917S-A, 917S-B, and 917S-C—all contain Ahp moiety, in fVIIa-sTF at 100 μg/mL. All of them gave negative inhibitory activity in fVIIa-sTF at 100 μg/mL.

Microviridin B is specific against thrombin. However, some reported microviridins—microviridins D to F [37]—do not inhibit thrombin. The presence of indole moiety, which is absent to other microviridins mentioned, may be the possible active thrombin scaffold in microviridin B.

## 3. Experimental Section

### 3.1. Laboratory Culture of M. aeruginosa K139

The *M. aeruginosa* K139 cyanobacterium was collected from Lake Kasumigaura, Ibaraki, Japan and was cultured in the laboratory with a CB medium [38]. The culture was transferred to a 10-mL CB medium and left for two weeks under continuous 24-h daylight. It was scaled up to 300 mL and left for two weeks before being further upscaled to 10-L CB medium. The cyanobacteria cells were harvested after two months. The algal cells were centrifuged in a Kubota 7000 at 9000 rpm. It was lyophilized and kept at −30 °C until use.

### 3.2. Isolation of fVIIa-sTF and Thrombin Inhibitors from M. aeruginosa K139

Pre-treatment of *M. aeruginosa* K139 algal cells and reversed-phase solid-phase extraction (ODS-SPE) involved the isolation of three compounds—aeruginosin K139, micropeptin K139, and microviridin B—which were patterned using the procedure developed by Nakano and Harada [30] with modifications. An 8.5 g of previously cultured and lyophilized algal cells were added with 300 mL of 5% CH_3_COOH (3×), homogenized for 30 min using a magnetic stirrer, and centrifuged for 5 min in the Kubota 5920 at 4000 rpm. The supernate was filtered in GF/C (Whatman^TM^, GE Healthcare UK, Limited, Buckinghamshire HP7 9NA, UK). The filtrate was flushed onto a Sep-Pak^®^ C18 35 mL Vac cartridge (Waters, Oasis, Ireland) sequentially preconditioned with 35 mL of MeOH, 50% MeOH, and H_2_O. It was eluted with 35 mL of 20% MeOH, 60% MeOH, and MeOH. Each band was collected separately (Supplementary Materials 1). The yellow band 2 contained the aeruginosin K139 (102.0 mg, crude) with *m/z* 603 [M + H]^+^ in LC-MS. The ODS-SPE was clinched with MeOH, which eluted micropeptin K139 and microviridin B (79.5 mg, crude).

### 3.3. Thin Layer Chromatography (TLC)

The developing solvent 65:35:10 CHCl_3_:MeOH:H_2_O (lower phase) was prepared before the TLC experiment. Proportionate amounts of CHCl_3_, MeOH, and H_2_O were mixed in a separatory funnel. The resulting mixture was left to stand for 10–30 min. The lower phase was drawn out and used as an eluent in the experiment. Adequate amounts of isolates were dissolved in a solvent above. Solutions were spotted on the pre-coated silica TLC plate (Kieselgel 60/Kieselgur F_254_, Merck, Darmstadt, Germany), air-dried, and developed in the TLC tank with developing solvent. After which, the developed plate was viewed under UV 254 nm. The plate was detected with I_2_ crystals.

### 3.4. Open Column Chromatography

The column chromatography (CC) solvent 65:25(35):10 CHCl_3_:MeOH:H_2_O, lower phase was also prepared similarly as the TLC developing solvent in Section 3.3.

#### 3.4.1. Aeruginosin K139

The 68.5 mg of the yellow band 2 (Supplementary Materials 1), aeruginosin K139-containing extract from the 60% MeOH extract underwent column chromatography using silica gel (BMW-300, Fuji Sylysia Chemical Ltd., Kasugai, Japan). The 60% MeOH extract was dissolved in 30:20:4 CHCl_3_:MeOH:H_2_O, and concentrated in vacuo using a rotary evaporator (Eyela) at 40 °C. A 20.5 mg of a soluble extract was loaded onto the pre-conditioned silica gel column and eluted with 65:35:10 CHCl_3_:MeOH:H_2_O, lower phase. Ten grams of silica gel was loaded onto the column, 50 cm in length, with a bed volume of 110 mL. A flow rate of 1 drop/12 s for 30 min collected a semi-pure aeruginosin K139. We repurified the aeruginosin K139-containing fraction using the same process. We isolated a tailed–spot compound in TLC with *m/z* 603 and *m/z* 635 in LC-MS (Supplementary Materials 3). Indeed, we were able to isolate 1.19 mg of aeruginosin K139. Complete assignment of chemical shifts have been discussed in this paper.

#### 3.4.2. Micropeptin K139 and Microviridin B

The MeOH ODS-SPE fraction, 79.7 mg, was loaded onto the silica gel column with 110-mL bed volume. A 65:25:10 CHCl_3_:MeOH:H_2_O (lower phase) was used as an eluent to isolate microviridin B and micropeptin K139. The eluent was adjusted to a flow rate of 1 drop/14 s and was collected for 20 min before replacement with a new test tube. A 10.45 mg mixture of microviridin B and micropeptin K139 was eluted and further purified using the same open column chromatography conditions with a flow rate of 1 drop/20 s for 30 min (for each test tube) to obtain 2.55 mg microviridin B, and 3.46 mg micropeptin K139.

### 3.5. LC-MS and LC-MS/MS

LC-MS experiments were performed in an LCQ decaXP Plus iontrap mass spectrometer (Thermo Finnigan/Thermo Scientific, San Jose, CA, USA) with an Agilent 1100 series liquid chromatography system. The mass spectrometer was set to 250 °C capillary temp unless otherwise specified. The accurate mass measurement was performed in a Sciex TripleTOF 6600 (AB Sciex, Framingham, MA, USA) quadrupole-time-of-flight (Q-TOF) mass spectrometer coupled with a Shimadzu Nexera XR LC system (Shimadzu Scientific Instruments, Inc., Columbia, MD, USA): Nexera XR LC-20AD_XR_ liquid chromatograph, DGU-20A_3R_ (Shimadzu Scientific Instruments, Inc., Columbia, MD, USA) degassing unit, CBM-20A communications Bus module, Nexera XR SIL-20AC_XR_ autosampler, and a CTO-20AG Prominence (Shimadzu Scientific Instruments, Inc., Columbia, MD, USA) column oven.

Aeruginosin K139 was dissolved in MeOH to make 1 mg/mL solution. Eighty microliters (80 µL) of water was added to the vial insert (Supelco, North Harrison Road, Bellefonte, PA, USA), and 20 µL of 1 mg/mL was pipetted and transferred to the vial insert to make 100 µg/mL of 20% MeOH. Ten microliters (10 µL) of 100-µg/mL solution was injected to the mass spectrometer. The ions were monitored in a solvent gradient from 20% MeOH with 0.1% HCOOH to 70% MeOH with 0.1% HCOOH over 60 min (Supplementary Materials 3) in a Super-ODS (TSKgel TOSOH, Tokyo, Japan) column 50 × 2.0 mm. The tautomerized aeruginosin K139 displayed retention times (t*_R_*) of 6.6, 7.7, 8.4, and 9.5 min. The same LC-MS condition was applied in ABSciex TripleTOF 6600 to obtain the HR-MS spectrum of aeruginosin K139 (Supplementary Materials 4).

The same sample preparation as aeruginosin K139 was undertaken for the LC-MS analysis of micropeptin K139. However, the solvent gradient in the LC system was extended to 90% MeOH with 0.1% HCOOH over 60 min (Supplementary Materials 13b). Micropeptin K139 eluted at t*_R_* 30.6 min with *m/z* 987. Similar gradient conditions and LC-MS parameters were applied to obtain the HR-MS of micropeptin K139 (Supplementary Materials 13d). The LC-MS/MS spectrum of micropeptin K139 was achieved in 10% MeCN containing 0.1% HCOOH to 100% MeCN with 0.1% HCOOH for 60 min [12] (Supplementary Materials 13c). A 10-µL and 25-µL injection volume of 100 µg/mL 10% MeCN solution were injected for MS and MS/MS, respectively. The capillary temp of the mass spectrometer was set to 200 °C, Collision-induced dissociation (CID) 30, isolation width 3, and mass range 325–1000.

Microviridin B was run in the LC-MS simultaneously with micropeptin K139. The solvent gradient was from 5% MeCN with 0.1% HCOOH to 100% MeCN with 0.1% HCOOH over 60 min. A 5 µL of 100 µg/mL of microviridin B was injected into the mass spectrometer, with t*_R_* 20.7 min, *m/z* 1723 and *m/z* 871.

### 3.6. 1D-NMR and 2D-NMR

The ^1^H-NMR, ^13^C-NMR data of the isolated compounds—aeruginosin K139, microviridin B, and micropeptin K139—from *M. aeruginosa* K139 were obtained by DMSO-*d*_6_ and CD_3_OD. A 1.19 mg of aeruginosin K139 was analyzed in Bruker 600 MHz in DMSO-*d*_6_. The ^1^H-NMR experiment of microviridin B was determined both in DMSO-*d*_6_ and CD_3_OD using 500 MHz JEOL JNM ECA-500. The micropeptin K139 was dissolved in DMSO-*d*_6_ and analyzed in Bruker Avance III HD 600 MHz (Supplementary Materials 13a).

The 2D data for aeruginosin K139 were analyzed using DMSO-*d*_6_ for HSQC, DQF-COSY, and ROESY (Supplementary Materials 7, 8 and 11). HMBC was analyzed using C12-DMSO-*d*_6_ at 30 °C and 50 °C (Supplementary Materials 9 and 10). At 50 °C, the dynamics of the compound was very rapid, and the viscosity of the solvent got low at high temperature. In this, we were able to obtain a clean HMBC spectrum.

### 3.7. Hydrolysis and Advanced Marfey Analysis

The stereochemistry of Leu was done byAdvanced Marfey’s method [32,33]. Aeruginosin K139 (200 µg) was dissolved in 6 M HCl (500 µL) and hydrolyzed as previously described [16,32]. The hydrolysate was concentrated in vacuo at 40 °C, and was subjected to advanced Marfey’s experiment. The FDLA solution was analyzed by LCQDecaXP with the same LC system in Section 3.5 using the gradient elution previously described by Anas et al. [16], using TOSOH Super ODS (TSKgel) column 100 × 2 mm, 2 mm id. Retention times (t*_R_*, min): l-Leu 12.2; d-Leu 20.3; aeruginosin K139 hydrolysate 12.5.

### 3.8. FVIIa-sTF and Thrombin Assays

The fVIIa-sTF and thrombin assays have been previously described in previous papers [3,12,16]. The half maximal effective concentration (EC_50_) compounds isolated from *M. aeruginosa* K139 was done in three plates, with two replicates in each of plate. A 1 mg/mL EtOH solution was prepared and independently diluted to make 750, 500, 250, 100, 75, 50, 25, 10, 5.0, 2.5, and 1.0 μg/mL. The same experimental procedure was done in all fVIIa-sTF and thrombin assays as previously described [12]. A 20-μL solution was added in each well, in replicate, to make a final concentration of 100, 75, 50, 250, 100, 75, 50, 25, 10, 5.0, 2.5, and 1.0 μg/mL. The EC_50_ was calculated from GraphPad Prism 7^©^ [34], with 95% confidence.

## 4. Conclusions

Three compounds—aeruginosin K139, microviridin B, and micropeptin K139—isolated from the study displayed anticoagulant activity in thrombin and fVIIa-sTF assays. Aeruginosin K139 is a potent thrombin inhibitor with an EC_50_ of 0.66 µM. It also demonstrated fVIIa-sTF inhibitory activity at 166 µM. Microviridin B is a thrombin-specific inhibitor with an EC_50_ of 4.58 µM. Micropeptin K139 exhibited a favorable fVIIa-sTF inhibitory activity with an EC_50_ of 10.62 µM, with a thrombin/fVIIa-sTF ratio of 2.54. From this study, *M. aeruginosa* K139 is a new source of fVIIa-sTF and thrombin inhibitors. This study opens an avenue for arginine-containing compounds and their derivatives, linear peptides, and cyclic depsipeptides from cyanobacteria as a unique source of fVIIa-sTF inhibitors.

## Figures and Tables

**Table 1:** 1D and 2D-NMR data of aeruginosin K139.

Unit		^1^H	*J* (Hz)	HSQC	HMBC	Cosy Double Quantum Filter (DQF-COSY)	Rotational-Frame Nuclear Overhauser Effect SpectroscopY (ROESY)
Hpla	1			174.9			
	2	4.05	*dd* (3.4,7.2)	72.0	1	3a,3b	3b,5,9,2-Leu
	3a	2.66	*dd* (14.0,7.2)	39.0	1,9	2	3b,5,9
	3b	2.86	*dd* (14.0,3.4)		4	2	2,3a,5,9
	4			128.0	6,8		
	5,9	6.99	*d* (8.3)	130.4	5,7,9	6,8	2,3a,3b,6,8
	6,8	6.64	*d* (8.3)	114.6	7	5,9	5,9
	7			155.6	6,8		
	2-OH	8.70	*s*				
	7-OH	9.16	*s*				
Leu	1			166.0			5, Hpla-2
	2	4.66	*t* (8.4,8.9)	59.5		3,NH-Leu	5
	3	1.36	*m*	42.0		2,4	2
	4	1.35	*m*	24.5		3,5,5′	5
	5	0.89	*d* (6.2)	21.4	4	4	2,4,Choi 6-OH
	5′	0.83	*d* (6.2)	23.3	3,4,5	4	
	NH	7.41	*d* (8.1)		Hpla-1,1	2	
Choi	1			173.0			
	2	4.15	*t* (2.5,6.3)	59.5	Leu-1,7′	2,3a,3b	6-OH
	3a	1.70 (*eq*)	solvent overlap	29.5	1	2,3′	6
	3b	2.45 (*ax*)	solvent overlap			2,3′	
	3′	2.28	*m* (*broad*)	36.0		3a,3b,4a,4b,7′	5b,7′
	4a	1.50	*m*	20.0		3′	
	4b	1.80	*m*			3′,5	5b
	5a	1.43	*m*, overlap	26.0		4b,6	6-OH
	5b	1.45					3′,4b,6
	6	3.92	*broad*	64.0		5,7a	3a,5b,7b
	7a	2.90 (*eq*)	*inc dd*	33.7	5	6,7′	7′
	7b	1.66 (*ax*)	*s*			7′	6
	7′	4.05	*dd* (3.4,3.6)	54.0	2	3′,7a,7b	3′, 6-OH,7a
	6-OH	4.51	*broad s*				2, 5a,7′
Argal (cyclic)	2	5.79	*broad*	74.0	C=N	6-OH	4a,4b
	3	4.90/4.55	*m*	56.5	2	4a,4b	4a,5a
	4a	2.05	*m (broad)*	36.0	C=N	3,5a,5b	2,3,5a
	4b	2.27	*m (broad)*			3,5a,5b	2
	5a	1.45	*m*, overlap	26.0		4a,4b	3,4a,6a
	5b	1.65				4a,4b	6a
	6a	2.66			2		
	6b	3.07	*m*	40.5			5a,5b
	NH	8.12	*broad*		1-Choi, 2		
	C=NH	7.35	*broad*		C=N		
	C=NH	8.53	*s*		C=N		
	C=N			158.0			
	2-OH	8.15	*broad s*				

**Table 2:** EC_50_ of compounds from *M. aeruginosa* K139.

Compounds	FVIIa-sTF: EC_50_, μM *	Thrombin: EC_50_, μM *	Thrombin/FVIIa-sTF EC_50_ Ratio
APMSF	19.07	2.10	0.11
Leupeptin	13.97	18.31	1.31
Aeruginosin K139	~166	0.66	0.004
Micropeptin K139	10.62	26.94	2.54
Microviridin B	(-)	4.58	NA

Legend:* 95% confidence using GraphPad Prism 7 [34]; NA: not applicable.

## Supplementary Materials

The following are available online at www.mdpi.com/1660-3397/15/9/275/s1.
