# Supplementary material for: FVIIa-sTF and Thrombin Inhibitory Activities of Compounds Isolated from Microcystis aeruginosa K-139
Source: Mar Drugs. 2017 Aug 30;15(9):275. doi: 10.3390/md15090275 (PMC5618414; doi:10.3390/md15090275)
Supplement: Supplementary file 1 [file marinedrugs-15-00275-s001.pdf]

Mar. Drugs **2008**, *6*, 1-x manuscripts

FVIIa-sTF and Thrombin Inhibitory Activities of Compounds Isolated from *Microcystis aeruginosa* K-139

Andrea Roxanne J. Anas[1,*], Akane Mori[1], Mineka Tone[1], Chiaki Naruse[1], Anna Nakajima[1], Hirohiko Asukabe[1], Yoshiaki Takaya[1], Susumu Y. Imanishi[1], Tomoyasu Nishizawa[2], Makoto Shirai[2] and Ken-ichi Harada [1, 3, *]

1 Faculty of Pharmacy, Meijo University, Tempaku, Nagoya 468-8503, Japan 120973255@ccalumni.meijo-u.ac.jp (A.M.); 110973437@ccalumni.meijo-u.ac.jp (M.T.); 100973149@ccalumni.meijo-u.ac.jp (C.N.); g0973345@ccalumni.meijo-u.ac.jp (A.N.); asukabe@bj8.so-net.ne.jp (H.A.); ytakaya@meijo-u.ac.jp (Y.T.); susima@meijo-u.ac.jp (S.Y.I.)

2 College of Agriculture, Ibaraki University, Ami, Ibaraki 300-0393, Japan tomoyasu.nishizawa.agr@vc.ibaraki.ac.jp (T.N.), makoto.shirai.kanoha@vc.ibaraki.ac.jp (M.S.)

3 Graduate School of Environmental and Human Sciences, Meijo University, Tempaku, Nagoya 468-8503, Japan

* Author to whom correspondence should be addressed; E-Mails: anasaroj@meijo-u.ac.jp (A.R.J.A.); kiharada@meijo-u.ac.jp (K.I.H.); Tel/Fax: +81-52-839-2720 (A.R.J.A.& K.I.H.)

Supplementary Information

List of Supplementary Information

Supplementary information

1. Separation of aeruginosin K139 from micropeptin K139 by reversed-phase solid-phase extraction (ODS-SPE), *3*.
2. LC-MS spectra of *M. aeruginosa* K-139-MeOH ODS-SPE fraction, *3*.
3. The LC-MS spectra of aeruginosin K139, *4*.
4. HR-MS spectrum of aeruginosin K139, *4.*
5. ^{1}H-NMR of aeruginosin K139 referenced to TMS (0.0 ppm), 600 MHz, *5*.
   5a. ^{1}H-NMR of aeruginosin K139 referenced to TMS (0.0 ppm) from 5.0 ppm to 0.01 ppm, 600 MHz, *5*.
   5b. ^{1}H-NMR of aeruginosin K139 referenced to TMS (0.0 ppm) from 8.7 ppm to 6.50 ppm, 600 MHz, *5*.
6. ^{13}C-NMR of aeruginosin K139 in DMSO-d_6 at 30°C referenced to TMS (0.0 ppm) from 135 ppm to 20 ppm, 600 MHz, *6*.
7. HSQC of aeruginosin K139 in DMSO-d_6 referenced to TMS (0.0 ppm) from 9.0 ppm to 0.01 ppm and 160 ppm to 0.01 ppm, 600 MHz, *6*.
8. DQF-COSY of aeruginosin K139 in DMSO-d_6 referenced to TMS (0.0 ppm) from 9.0 ppm to 0.01 ppm, 600 MHz, *7*.
9. HMBC of aeruginosin K139 in DMSO-d_6 at 30°C referenced to TMS (0.0 ppm) from 9.0 ppm to 0.01 ppm, 170 ppm to 0.01 ppm, 600 MHz, *7*.
10. HMBC of aeruginosin K139 in DMSO-d_6 at 50°C referenced to TMS (0.0 ppm) from 9.0 ppm to 0.01 ppm, 170 ppm to 0.01 ppm and 200 ppm to 0.01 ppm, 600 MHz, *8*.
11. ROESY of aeruginosin K139 in DMSO-d_6 referenced to TMS (0.0 ppm) from 9.0 ppm to 0.01 ppm, 600 MHz, 8.
12. Thin layer chromatography (TLC) profiles of MeOH ODS fractions with TLC pure micropeptin K139, and microviridin B after three open column chromatographies, *9*.

13:13a. ^{1}H-NMR of micropeptin K139 referenced to TMS, 600 MHz, *9*.
   13b. Liquid chromatography-mass spectrum (LC-MS) of micropeptin K139, *10*.
   13c. The LC-MS/MS spectra of micropeptin K139, *10*.
   13d. HR-MS spectrum of micropeptin K139, *11*.

14: 14a. ^{1}H-NMR of microviridin B referenced to CD_3OD (3.30 ppm), *11*.
   14b. ^{1}H-NMR of microviridin B referenced to DMSO-d_6 (2.49 ppm), *12*.
   14c. Liquid chromatogram-mass spectrum (LC-MS) of microviridin B, *12*.

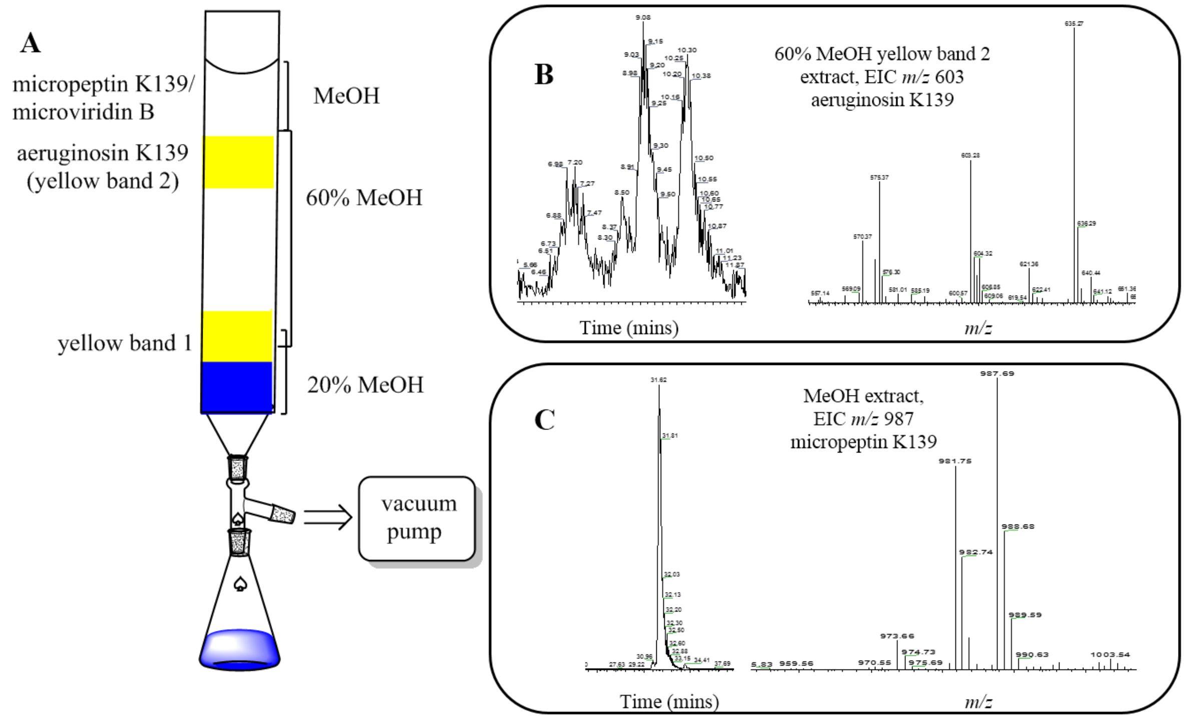

Supplementary information 1. Separation of aeruginosin K139 from micropeptin K139 by reversed-phase solid-phase extraction (ODS-SPE). A). ODS-SPE cartridge (35 mL) with eluting solvents 20% MeOH, 60% MeOH and MeOH; B). Extracted Ion Chromatogram (EIC) of yellow band 2 with *m/z* 603; C). EIC of MeOH extract with *m/z* 987. The solvent gradient used for liquid chromatography-mass spectrometry (LC-MS) was 20% MeOH with 0.1% HCOOH to 70% MeOH with 0.1% HCOOH over 60 mins for aeruginosin K139. Moreover, the solvent system for micropeptin K139 was similar with the gradient used for aeruginosin K139 but extended to 90% MeOH with 0.1% HCOOH, Super-ODS (TSKgel TOSOH Tokyo, Japan) column, 50 x 2mm, column temp 40˚C, capillary temp 250˚C

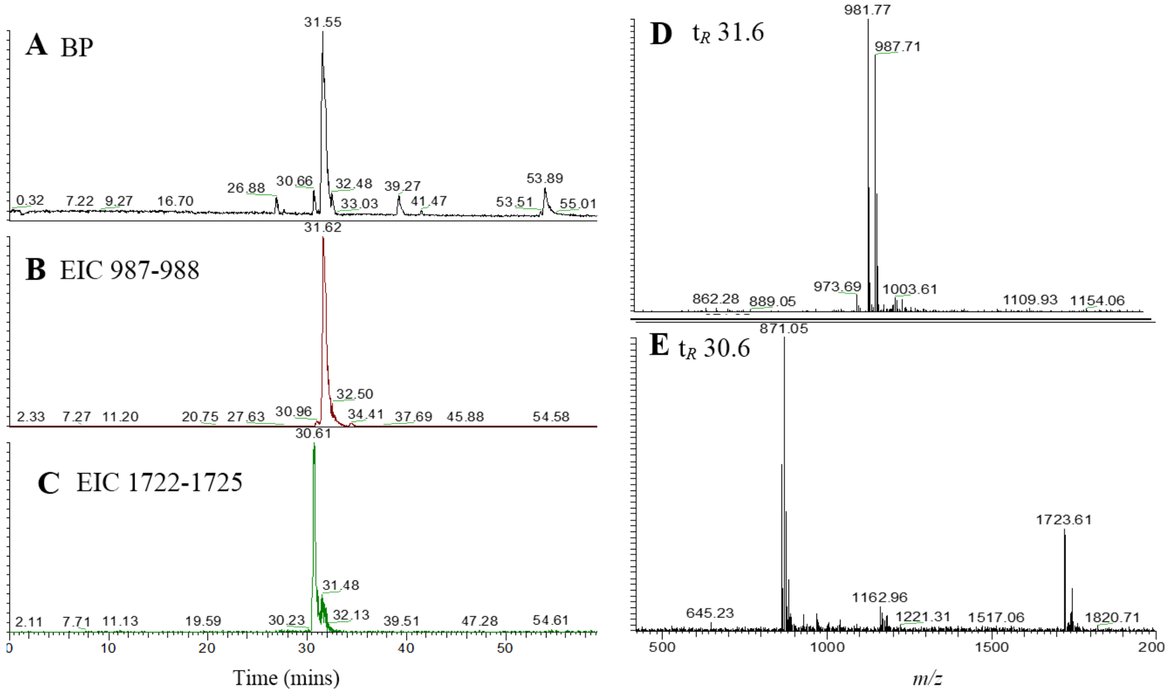

Supplementary information 2. LC-MS spectra of *M. aeruginosa* K-139-MeOH ODS-SPE fraction. A) Base Peak (BP); B). EIC *m/z* 987-988; C). EIC *m/z* 1722-1725; D). retention time (t_R, min) 31.6; E). D). t_R 30.6. The solvent gradient used for LC-MS was 15% MeOH with 0.1% HCOOH to 90% MeOH with 0.1% HCOOH over 60 mins.

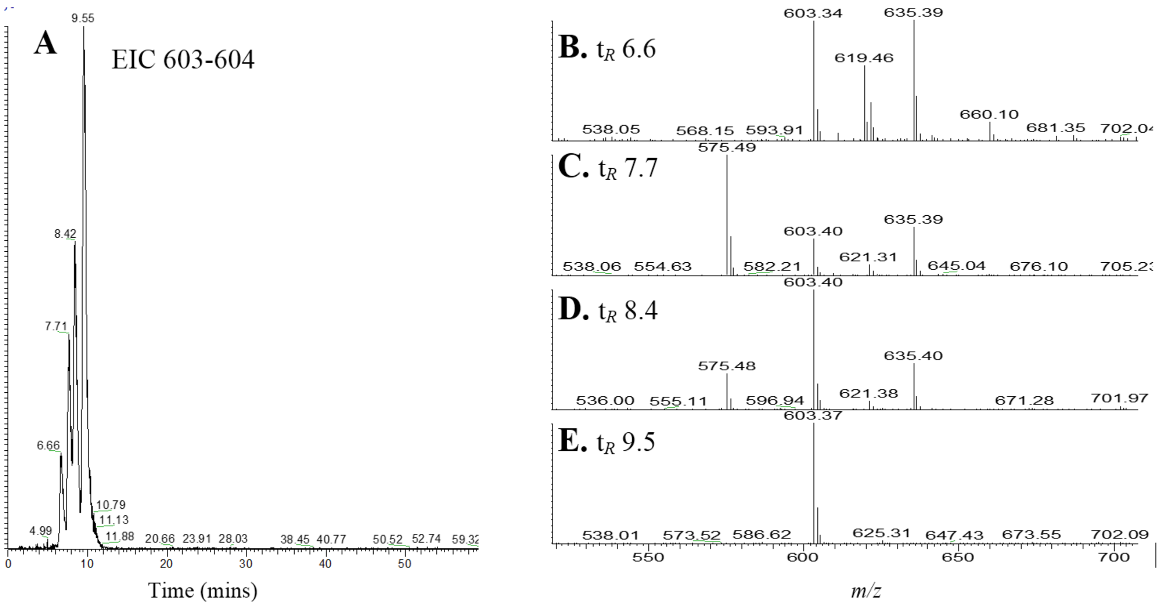

Supplementary information 3. The LC-MS spectra of aeruginosin K139. A). Extracted ion chromatogram (EIC 603-604) of aeruginosin K139 with solvent gradient from 20% MeOH with 0.1% HCOOH to 70% MeOH with 0.1% HCOOH over 60 mins, Super-ODS 50 x 2.0 mm, capillary temperature 250˚C, injection volume 10 μL of 100 μg/mL; B). Mass spectrum (MS) of aeruginosin K139 with t_R 6.6 min; C). MS of aeruginosin K139 with t_R 7.7 min; D). MS of aeruginosin K139 with t_R 8.4 min; e). MS of aeruginosin K139 with t_R 9.5 min.

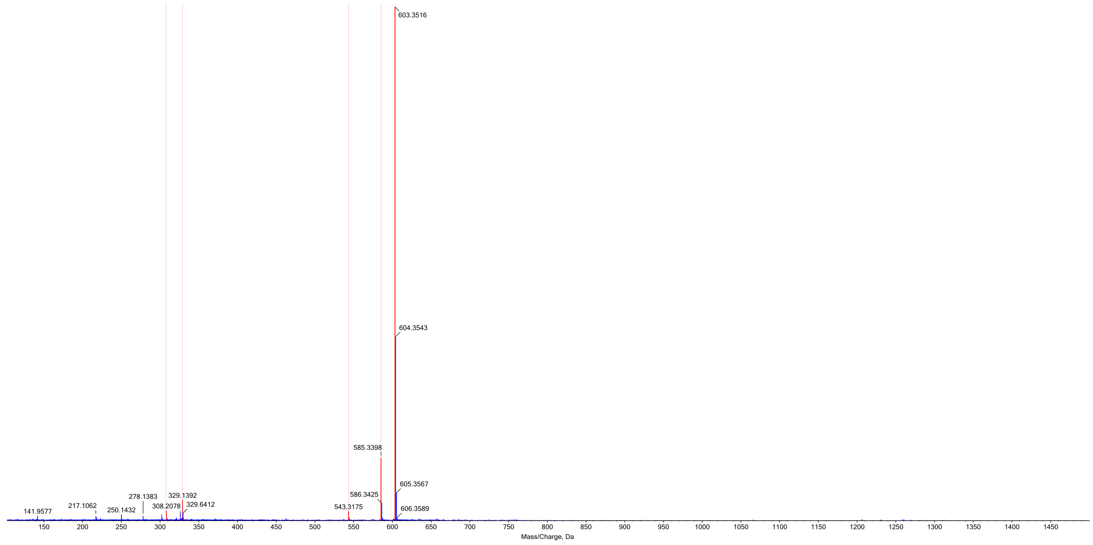

Supplementary information 4. HR-MS spectrum of aeruginosin K139.

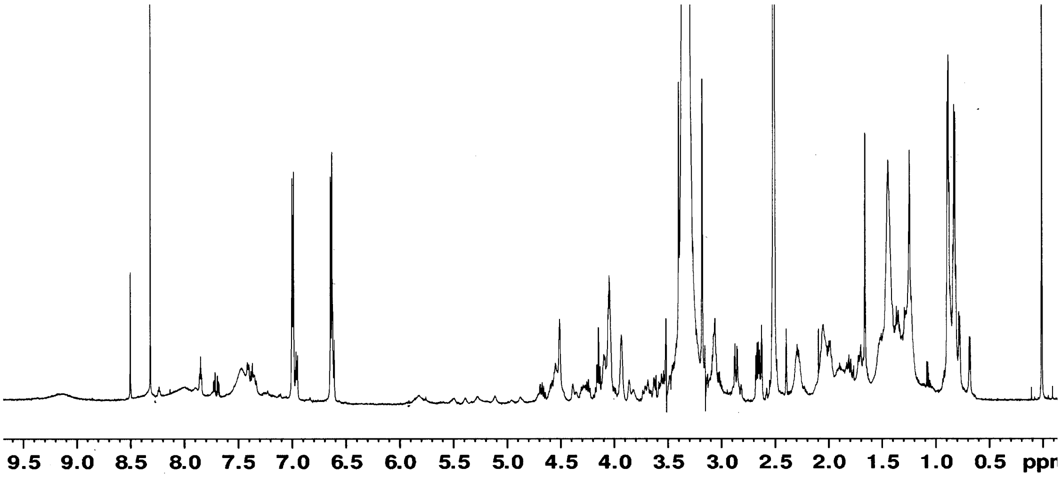

Supplementary Information 5. ^{1}H-NMR of aeruginosin K139 referenced to TMS (0.0 ppm), 600 MHz.

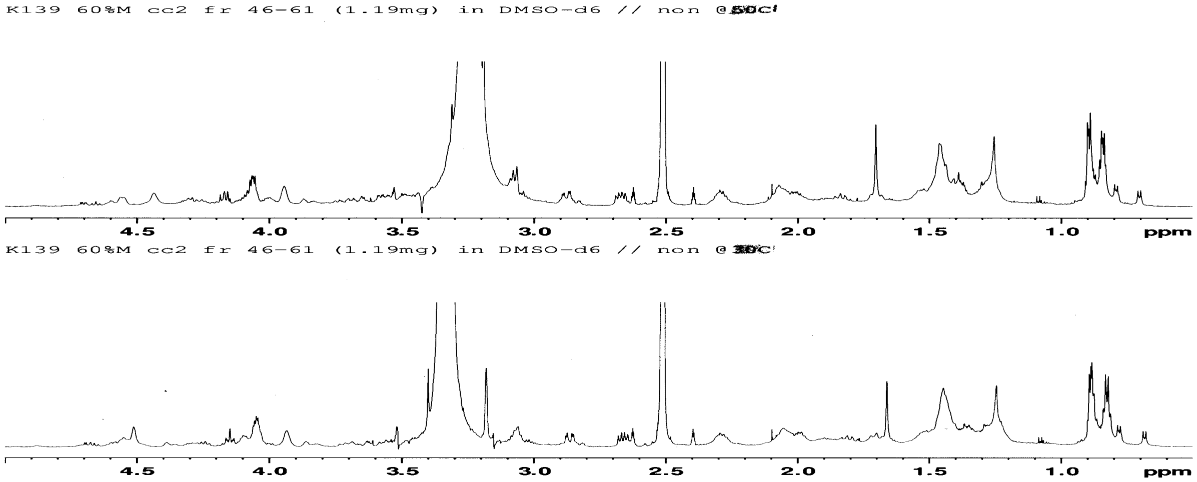

Supplementary Information 5a. ^{1}H-NMR of aeruginosin K139 referenced to TMS (0.0 ppm) from 5.0 ppm to 0.01 ppm, 600 MHz. Upper chromatogram (^{1}H-NMR of aeruginosin K139 at 50°C); lower chromatogram (^{1}H-NMR of aeruginosin K139 at 30°C).

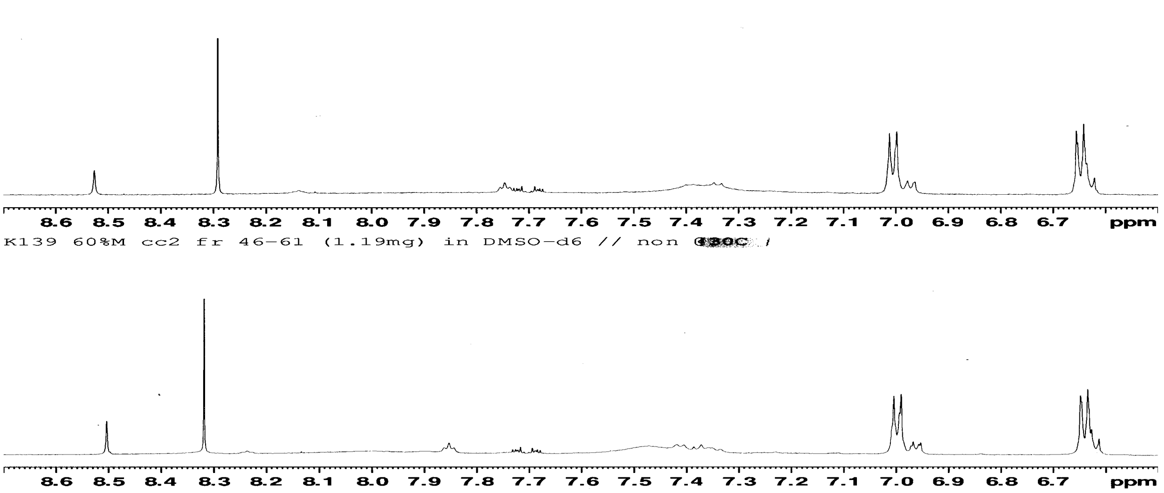

Supplementary Information 5b. ^{1}H-NMR of aeruginosin K139 referenced to TMS (0.0 ppm) from 8.7 ppm to 6.50 ppm, 600 MHz. Upper chromatogram (^{1}H-NMR of aeruginosin K139 at 50°C); lower chromatogram (^{1}H-NMR of aeruginosin K139 at 30°C).

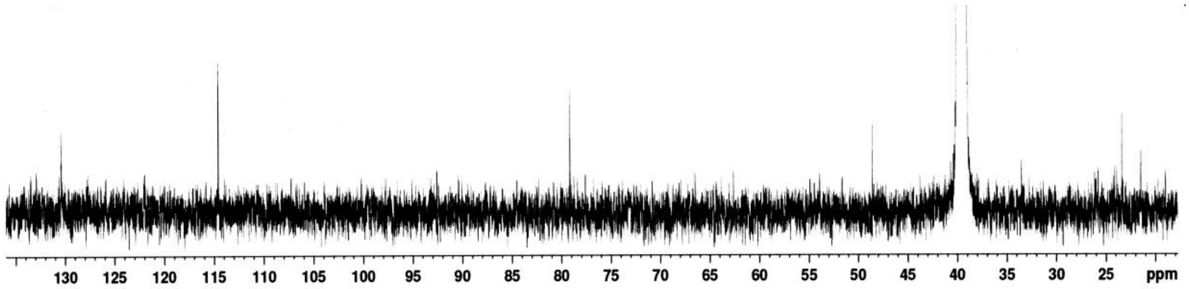

Supplementary Information 6. ^{13}C-NMR of aeruginosin K139 in DMSO-d_6 at 30°C referenced to TMS (0.0 ppm) from 135 ppm to 20 ppm, 600 MHz.

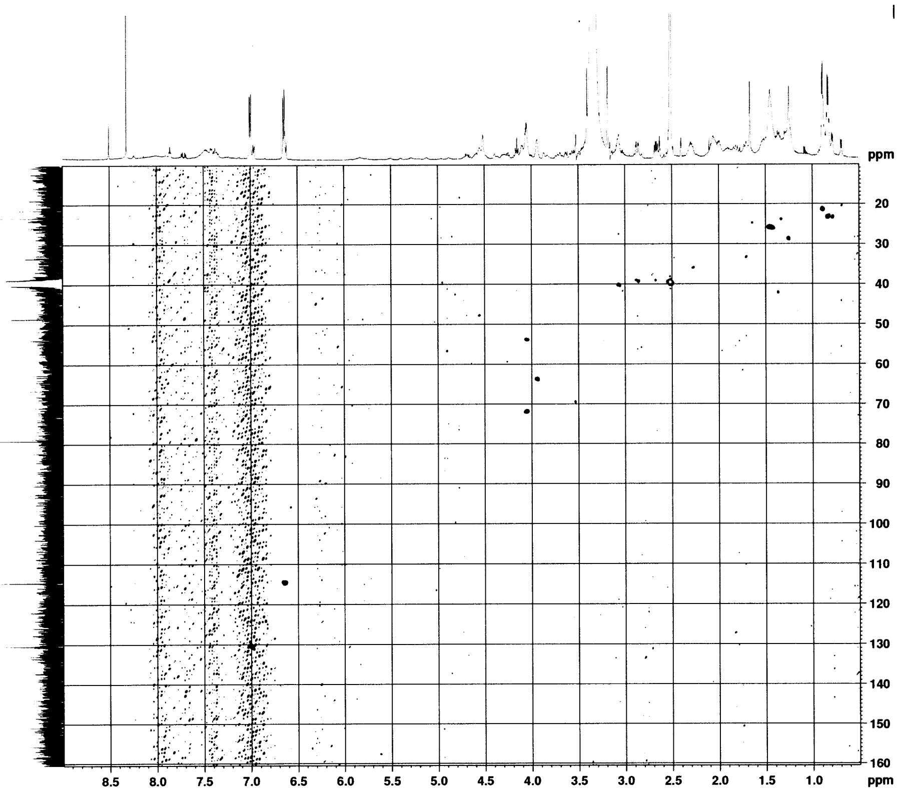

Supplementary Information 7. HSQC of aeruginosin K139 in DMSO-d_6 referenced to TMS (0.0 ppm) from 9.0 ppm to 0.01 ppm and 160 ppm to 0.01 ppm, 600 MHz.

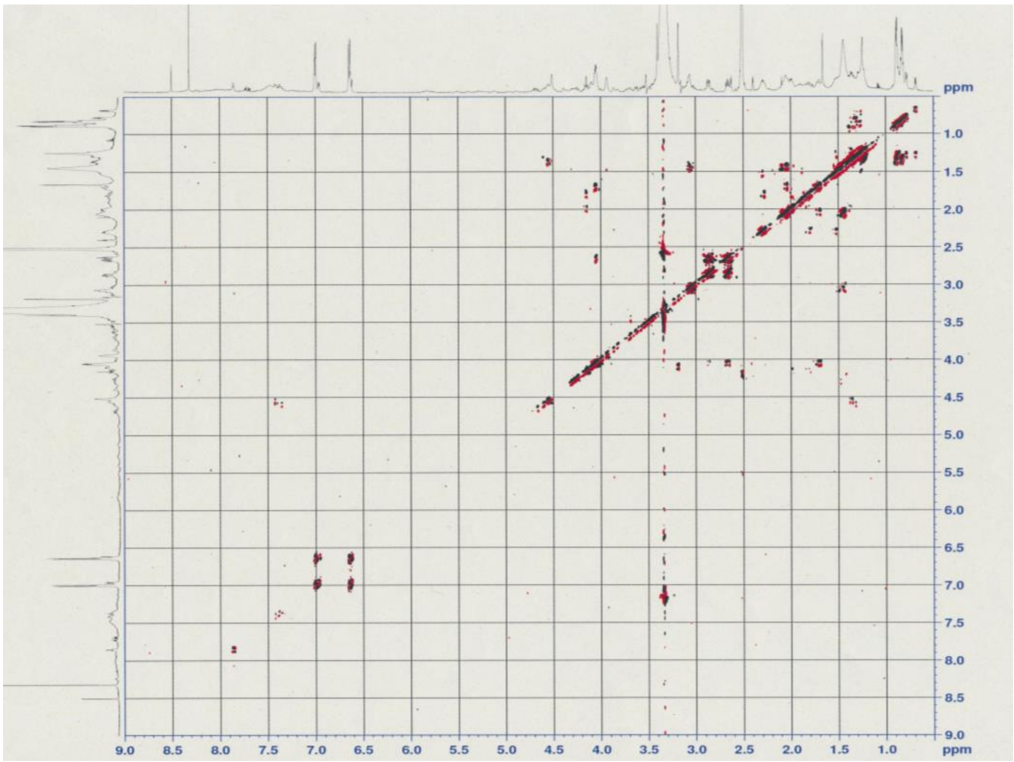

Supplementary Information 8. DQF-COSY of aeruginosin K139 in DMSO-d_6 referenced to TMS (0.0 ppm) from 9.0 ppm to 0.01 ppm, 600 MHz.

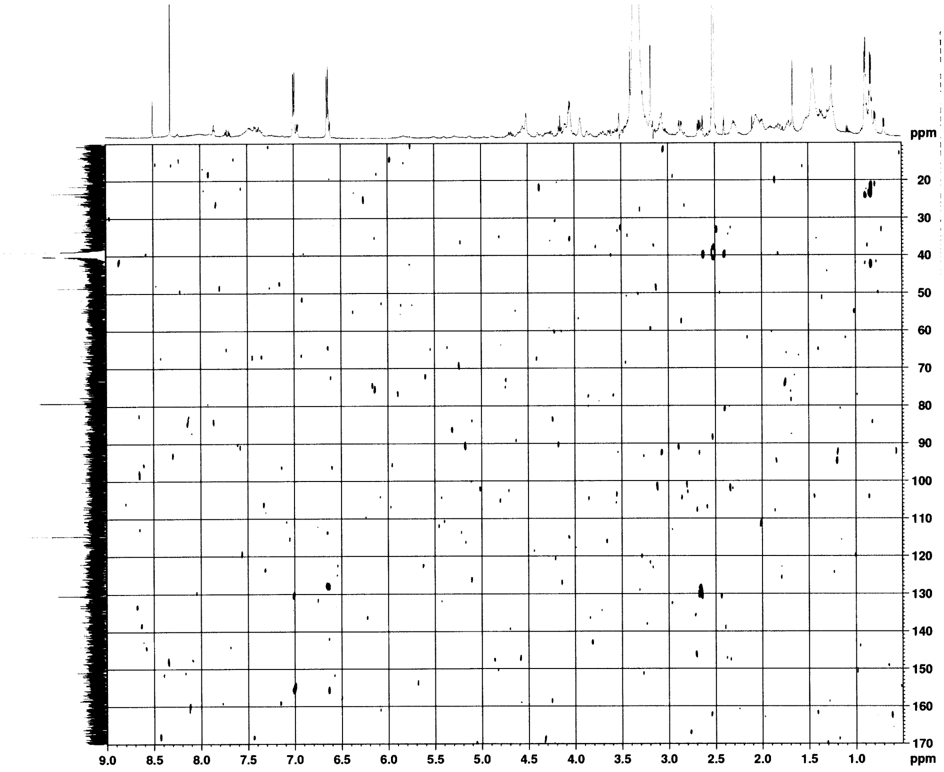

Supplementary Information 9. HMBC of aeruginosin K139 in DMSO-d_6 at 30°C referenced to TMS (0.0 ppm) from 9.0 ppm to 0.01 ppm, 170 ppm to 0.01 ppm, 600 MHz.

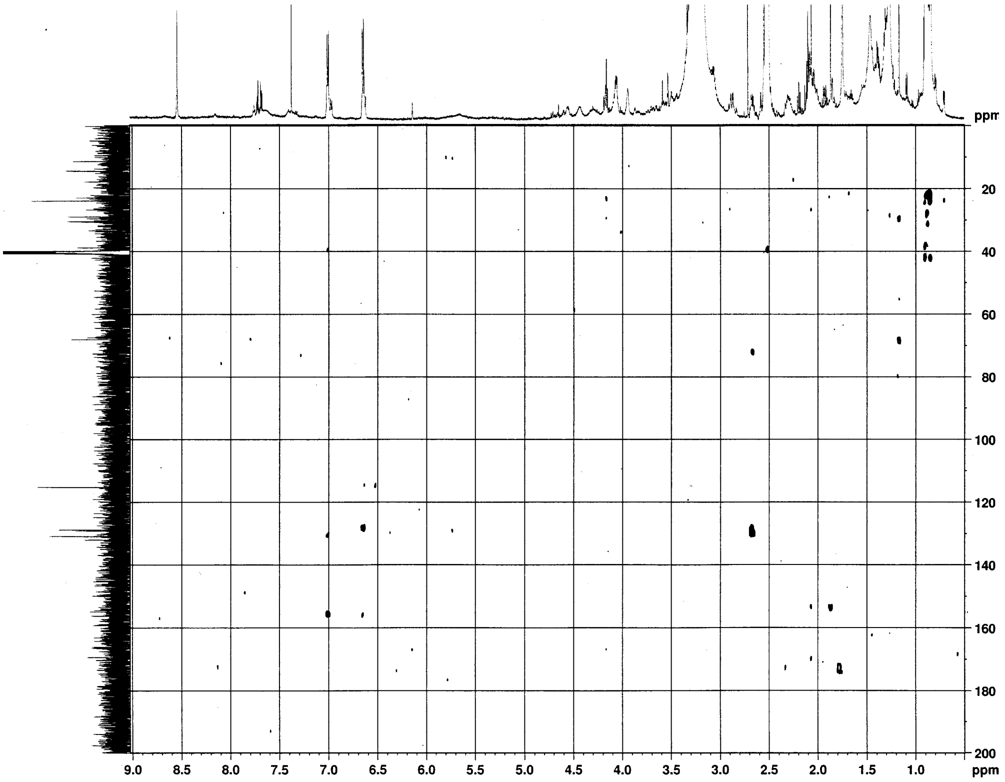

Supplementary Information 10. HMBC of aeruginosin K139 in DMSO-d_6 at 50°C referenced to TMS (0.0 ppm) from 9.0 ppm to 0.01 ppm, 170 ppm to 0.01 ppm and 200 ppm to 0.01 ppm, 600 MHz.

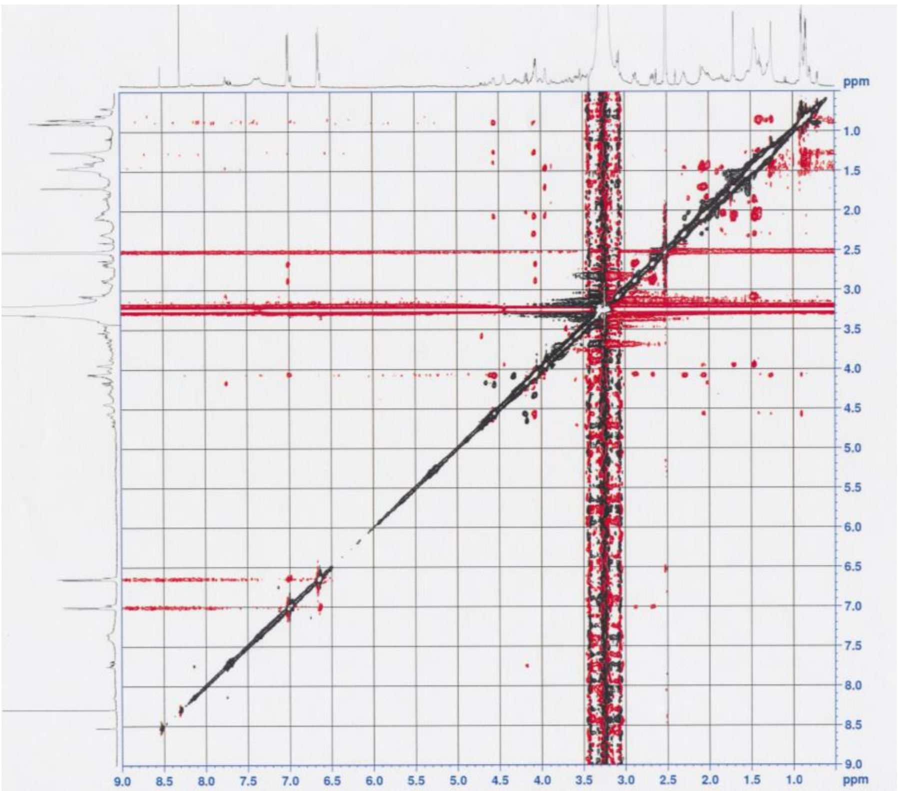

Supplementary Information 11. ROESY of aeruginosin K139 in DMSO-d_6 referenced to TMS (0.0 ppm) from 9.0 ppm to 0.01 ppm, 600 MHz.

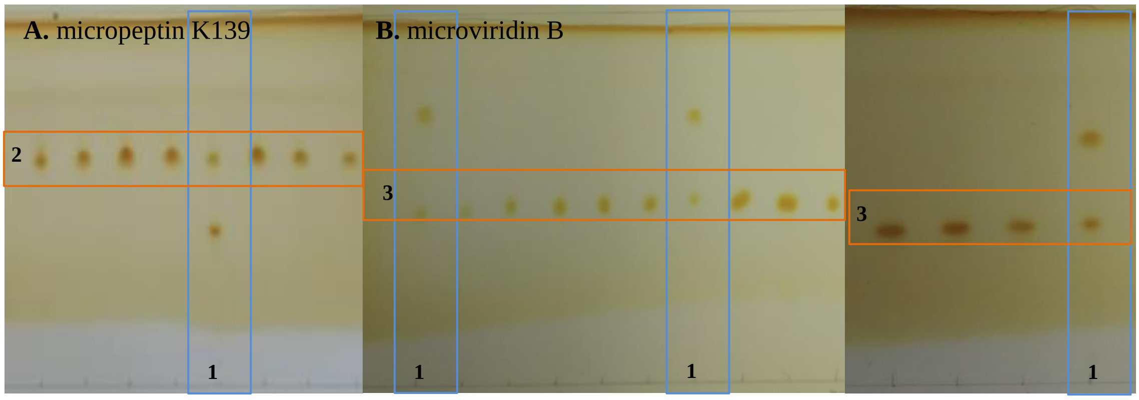

Supplementary information 12. Thin layer chromatography (TLC) profiles of MeOH ODS fractions with TLC pure micropeptin K139, and microviridin B after three open column chromatographies. A). micropeptin K139 plate; B). Microviridin B plates. The solvent system for TLC: 65:35:10 $CHCl_3$: MeOH: H_2O; detection in I_2 crystals. Legend: **1** (blue line), standard spots from second open column chromatography composed of microviridin B and micropeptin K139; **2**, micropeptin K139 spot isolated from third open column chromatography; **3**, microviridin B spot isolated from third open column chromatography using 65:25:10 $CHCl_3$:MeOH:H_2O (lower phase) as an eluent.

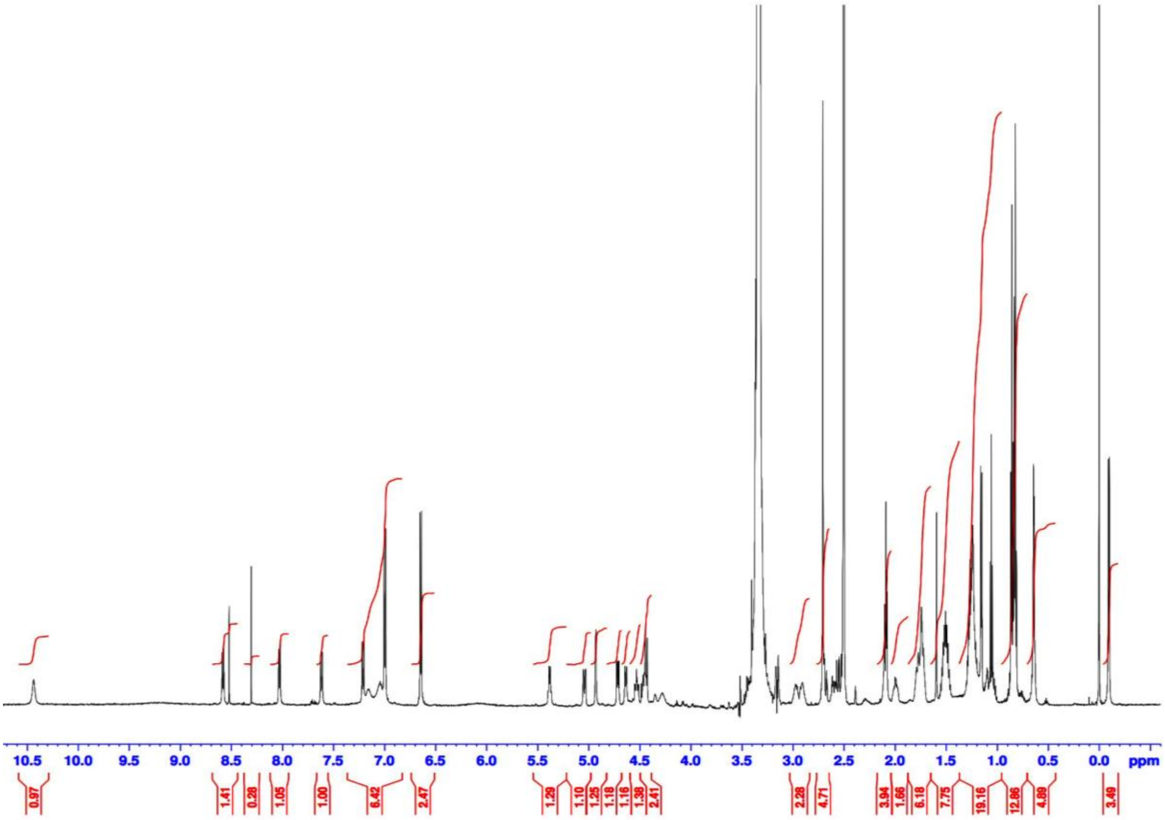

Supplementary Information 13a. ^{1}H-NMR of micropeptin K139 referenced to TMS, 600 MHz.

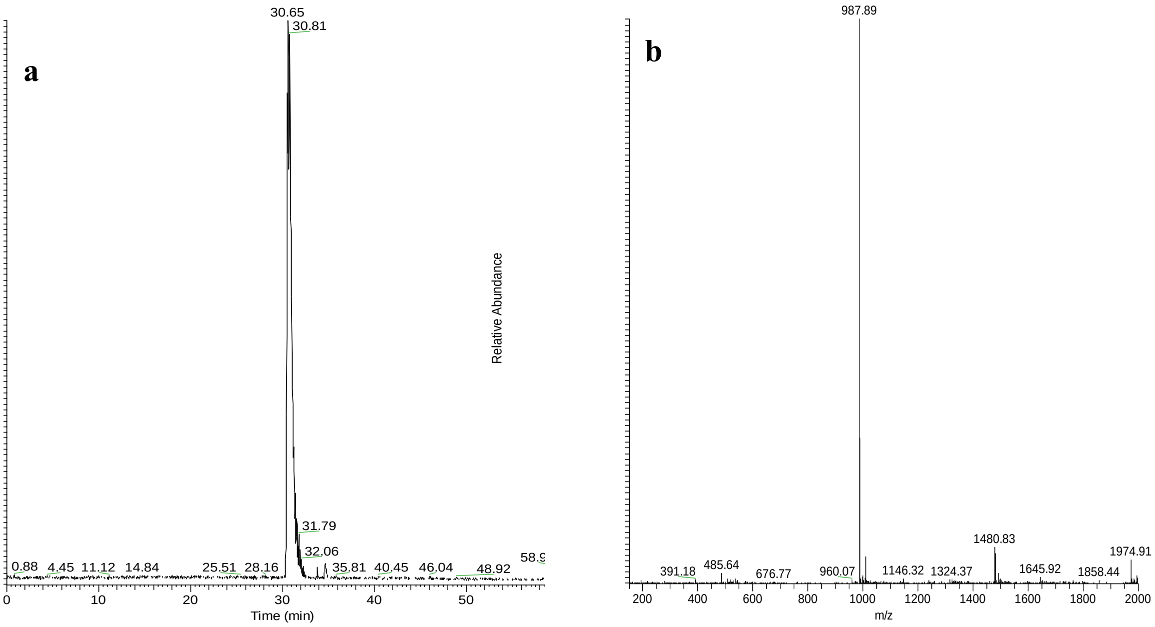

Supplementary Information 13b. Liquid chromatography-mass spectrum (LC-MS) of micropeptin K139. **a**). LC of micropeptin K139 in 20% MeOH with 0.1% HCOOH to 90% MeOH with 0.1% HCOOH over 60 mins using Super-ODS 2.0 x 50 mm column, capillary temp 250˚C, flowrate 200 μL/min, injection volume 10 μL of 100 μg/mL. **b**). Mass spectrum of micropeptin K139 with t_R 30.65 min.

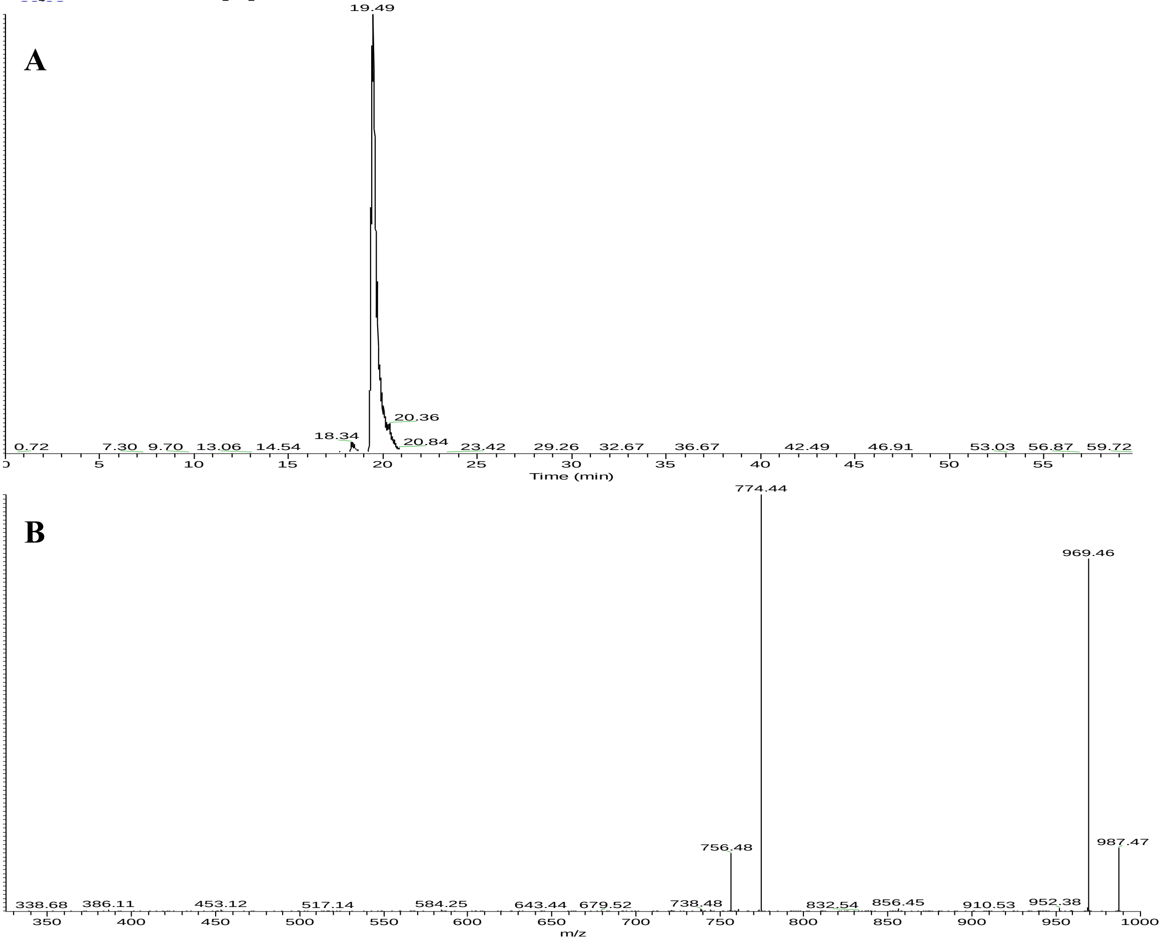

Supplementary information 13c. The LC-MS/MS spectra of micropeptin K139. A). The liquid chromatography-total ion chromatogram (LC-TIC) of micropeptin K139 with solvent gradient from 10% MeCN (0.1% HCOOH) to 100% MeCN (0.1% HCOOH) over 60 mins [15], TSKgel Super-ODS 50x2.0 mm, 25-μL injection volume of 100-μg/mL MeCN solution, t_R 19.4 min with *m/z* 987 $[M+H]^+$. B). MS/MS spectrum of parent ion *m/z* 987. Capillary temperature 200˚C, CID 30, isolation width 3; mass range 325-1000

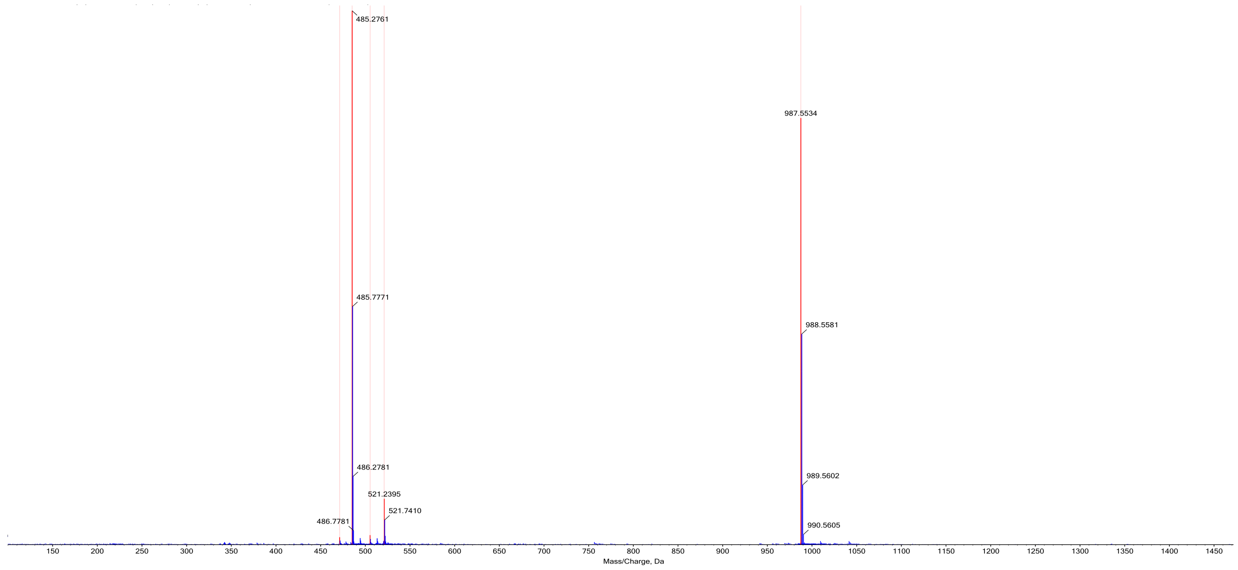

Supplementary information 13d. HR-MS spectrum of micropeptin K139.

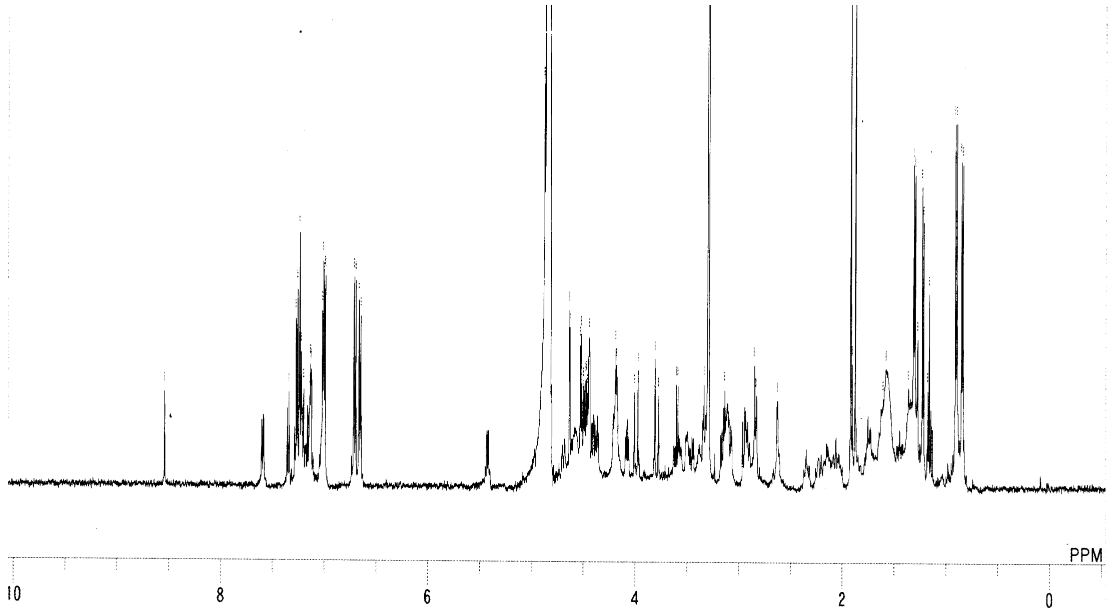

Supplementary Information 14a. ^{1}H-NMR of microviridin B referenced to CD_3OD (3.30 ppm).

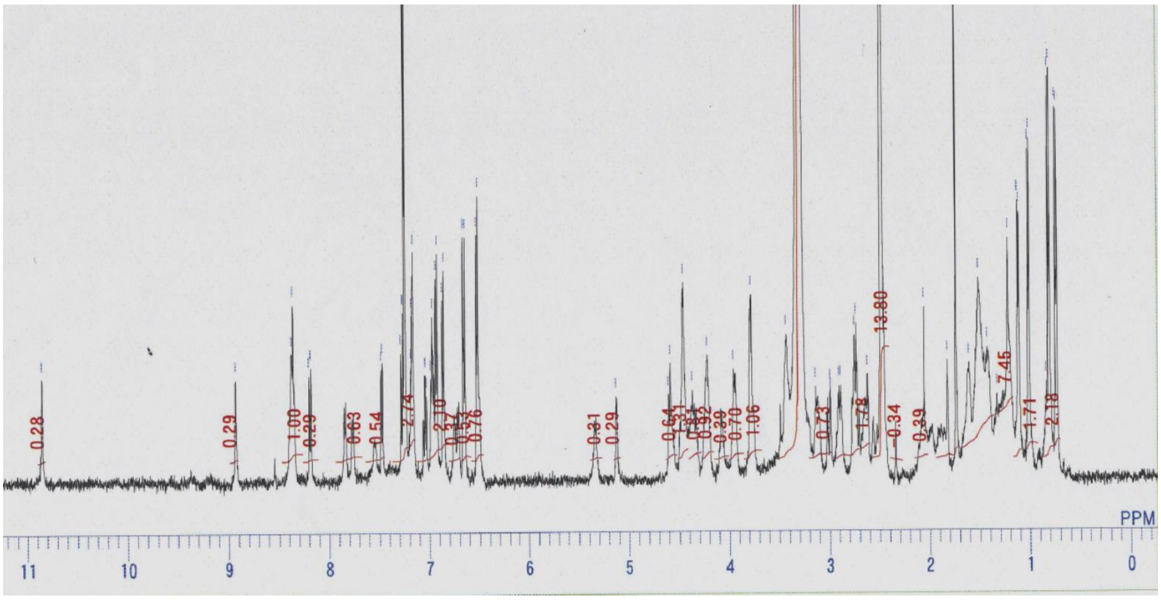

Supplementary Information 14b. ^{1}H-NMR of microviridin B referenced to DMSO-d_6 (2.49 ppm).

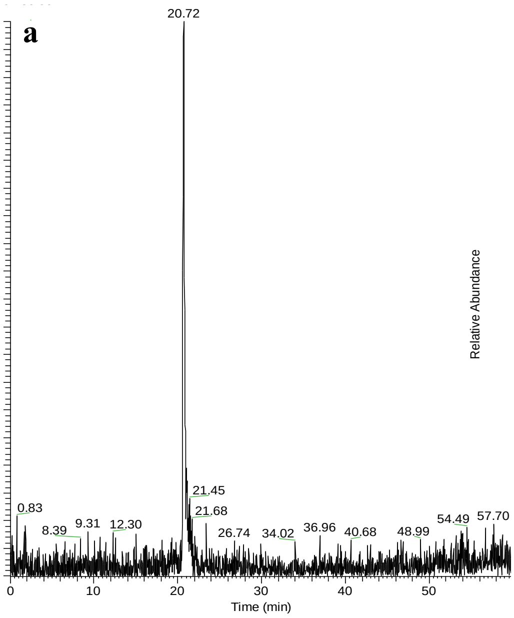

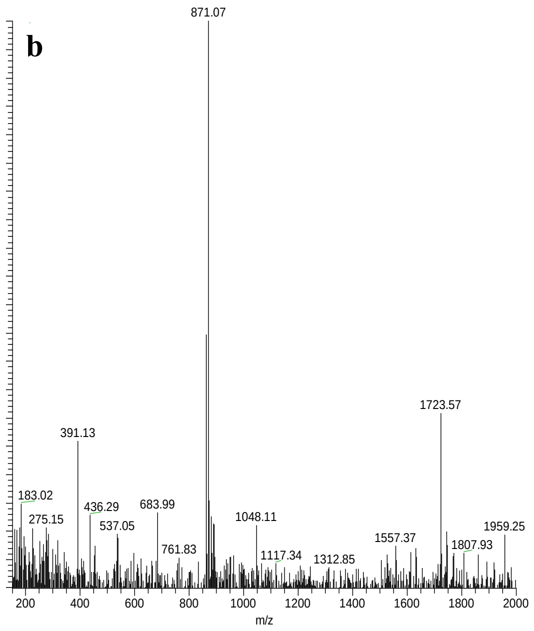

Supplementary Information 14c. Liquid chromatogram-mass spectrum (LC-MS) of microviridin B. **a**). LC of microviridin B in 5% MeCN-100% MeCN with 0.1% HCOOH over 60 mins using Super-ODS 2.0 x 50 mm column, capillary temp 250˚C, flowrate 200 μL/min, injection volume 5 μL of 100 μg/mL. **b**). Mass spectrum of microviridin B with t_R 20.7 min.
